# Impact of scale on the effectiveness of disease control strategies for epidemics with cryptic infection in a dynamical landscape: an example for a crop disease

**DOI:** 10.1098/rsif.2007.1019

**Published:** 2007-07-03

**Authors:** Christopher A Gilligan, James E Truscott, Adrian J Stacey

**Affiliations:** Department of Plant Sciences, University of CambridgeDowning Street, Cambridge CB2 3EA, UK

**Keywords:** epidemiological model, spatio-temporal dynamics, rhizomania, network model, soil-borne disease

## Abstract

We use a spatially explicit, stochastic model to analyse the effectiveness of different scales of local control strategies in containing the long-term, multi-seasonal spread of a crop disease through a dynamically changing population of susceptible crops in which there is cryptic infection. The model distinguishes between susceptible, infested and symptomatic fields. It is motivated by rhizomania disease on sugar beet in the UK as an exemplar of a spatially structured and partially asymptomatic epidemic. Our results show the importance of matching the scales of local control strategies to prevent intensification and regional spread of disease with the inherent temporal and spatial scales of an epidemic. A simple field-scale containment strategy, whereby the susceptible crop is no longer grown on fields showing symptoms, fails for this system with cryptic infection because the locally applied control lags behind the epidemic. A farm-scale strategy, whereby growers respond to the disease status of neighbouring farms by transferring their quota for sugar beet to farmers in regions of reduced risk, succeeds. We conclude that a soil-borne pathogen such as rhizomania could be managed by movement of susceptible crops in the landscape using a strategy that matches the temporal and spatial scales of the epidemic and which take account of risk aversion among growers. We show some parallels and differences in effectiveness between a ‘culling’ strategy involving crop removal around emerging foci and the local deployment of partially resistant varieties that reduce amplification and transmission of inoculum. Some relationships between the control of plant and livestock diseases are briefly discussed.

## 1. Introduction

The invasion of diseases at national and international scales is a frequent and recurring problem in crop and livestock production, in natural and semi-natural ecosystems and human populations. Recent and extensive examples include foot and mouth disease of domestic livestock in the UK (Ferguson *et al*. 2001*b*; Keeling *et al*. 2001) and Taiwan (Howard & Donnelly 2000), phocine distemper virus in North Sea seals (Swinton *et al*. 1998), Dutch elm disease in the UK (Swinton & Gilligan 1996), chestnut blight (Milgroom & Cortesi 2004) and sudden oak death in forest and woodland (Meentemeyer *et al*. 2004) in North America, citrus canker in Florida and South America (Gottwald *et al*. 2001), cassava mosaic disease in Africa (Legg 1999) and Asian soybean rust in Brazil. Global control strategies, such as the deployment of resistance genes and chemical control in crops or prophylactic vaccination of livestock, are seldom feasible for many of these emerging epidemics. There may be cost and logistical constraints or concerns for drug or pesticide resistance associated with widespread continual use of a control measure that imposes strong selection pressure on a pathogen. Localized deployment of control methods around emerging foci offers an alternative approach. Selection of an appropriate scale, however, to prevent invasion is complicated by the cryptic spread of infection ahead of the appearance of symptomatic individuals (Dybiec *et al*. 2004; Tildesley *et al*. 2006). It is also complicated by spatial and temporal heterogeneities in the spread of disease. These reflect the spatial arrangement of susceptible sites (fields or herds) in the landscape, associated with crop or animal husbandry, as well as seasonal disturbances, such as harvesting or culling. It follows that many emerging diseases appear to be loosely clustered, with localized spread around symptomatic sites and occasional long-distance transmission of infection to distant sites, often with periodic disturbances in epidemic spread. Simple theories for invasion and vaccination thresholds derived for well-mixed populations (Anderson & May 1991) are not applicable to these systems. Some of the shortcomings of using a model for homogeneous transmission to predict efficiency of control of the UK epidemic of foot and mouth disease are illustrated by Keeling *et al*. (2003). Instead, individual-based stochastic models for intervention of the spread of infection from farm to farm offer greater scope to optimize control strategies for the culling strategies in the 2001 epidemic (Ferguson *et al*. 2001*a*,*b*; Keeling *et al*. 2001, 2003) and for future reactive vaccination strategies (Tildesley *et al*. 2006).

The use of models to inform local and global strategies for the management and regional control of invading plant pathogens is less advanced than for animal diseases. The need for such models is apparent, however, as new disease threats emerge with changing cropping patterns in response to economic, climatic, political and social pressures (Gilligan in press). Here we use a spatially explicit, stochastic model (Stacey *et al*. 2004) to analyse the effectiveness of different scales of control strategies in containing the long-term, multi-seasonal spread of a crop disease through a dynamically changing population of susceptible crops in which there is cryptic infection. We use the spread of rhizomania disease on sugar beet in the UK as an exemplar of a highly, spatially structured and partially asymptomatic epidemic (Stacey *et al*. 2004). The analyses therefore address broad issues in matching the scales of control with the implicit spatial and temporal scales of the host–pathogen system. We consider the effectiveness of two strategies involving local management of disease around symptomatic sites. The first entails removal of susceptible and infected crops, whereby infected crops are destroyed and no more beet crops are grown in the vicinity. This type of control is analogous to culling practices used to control livestock diseases, except that ‘restocking’ is not permitted at the same site because soils remain infested for long periods. Specifically, we compare the effectiveness of field-scale policies with those that operate at larger scales. The problem therefore translates into an analysis of optimal local control, with the additional constraint of redistribution of susceptible crops within a heterogeneously infected landscape to maintain national cropping levels. The second type of control involves local deployment of partially resistant cultivars around symptomatic sites. Partially resistant varieties are analogous to partially effective vaccines in that they diminish but do not prevent the multiplication of inoculum. Local cultivation of partially resistant varieties therefore corresponds to a reduction in the strength of the connection between infested and susceptible farms.

Rhizomania is a soil-borne disease of sugar beet that first appeared in the UK in 1987, having spread through continental beet-growing regions in the preceding decades (Asher 1993). It is caused by beet necrotic yellow vein virus, which is transmitted by a ubiquitous soil-borne vector, the Plasmodiophoromycete, *Polymyxa betae* (Agrios 2005). The disease is typical of many economically important diseases; it is highly persistent once introduced and there is extensive dispersal through the landscape (within and between farms) before symptoms become apparent giving rise to sparse and cryptic patterns of infestation (Stacey *et al*. 2004). Sugar beet accounts for approximately 150 000 ha in the UK, with a fixed quota for production set by the industry. Prior to January 2002, there was a statutory containment policy operated by the then Ministry of Agriculture Fisheries and Food (MAFF, now the Department for Environment, Food and Rural Affairs, Defra). Rhizomania was declared to be a notifiable disease; extensive surveys were carried out annually. Any infected sugar-beet crops were destroyed *in situ* and further growth of sugar beet on affected fields was prohibited. This type of procedure is analogous to local culling of infecteds and susceptibles for livestock diseases. The risks of primary infection from external resources were reduced by imposing import restrictions on the entry of plant material, such as potatoes, that might carry soil from rhizomania regions and by strict policies to ensure that sugar-beet waste was not returned to arable land. The statutory field-scale strategy allowed individual farmers with symptomatic fields to maintain their output by growing sugar beet on other fields within their farm. A second farm-scale scheme was introduced by the sugar-beet industry, which controls the total area sown to the crop and the distribution of this quota among individual growers. Under the scheme, growers concerned about the risk of rhizomania on their farms could apply to donate some or all of their quota to other growers, thereby maintaining but redistributing the total acreage of susceptible crops. The scheme, known as stewardship, therefore introduces a break in connection between infested and susceptible farms. It differs from conventional culling of susceptibles because it preserves the population of susceptible hosts. Following the availability of partially resistant varieties of sugar beet (Scholten & Lange 2000) and intensive EU discussion, the field-scale containment policy was discontinued in January 2002. The stewardship scheme was withdrawn a little later, although growers are still able to buy and sell quotas. It is apparent, however, that partially resistant varieties permit amplification of inoculum. The numbers of fields exhibiting symptoms of rhizomania have continued to increase in reported occurrence in 2006 (Anonymous 2006). Accordingly, we revisit the strategies for local control. The analyses presented here are designed to answer the following five questions.

- Can the disease be managed by movement of susceptibles in the landscape?
- How is this influenced by different degrees of risk aversion among growers?
- What are the characteristic spatial and temporal scales of local control for global management of disease in the landscape?
- How are these scales influenced by cryptic infection?
- How does the local deployment of partially resistant varieties impact on the control strategies?

## 2. Methods

### 2.1 Model

Annual cycles of rhizomania epidemics are divided into two distinct periods: an amplification phase, as soil-borne viruliferous spores of the vector infect the host and multiply during the growing season, and a dispersal phase mainly on agricultural machinery at harvest. This yields a stochastic, spatially explicit model for the spread of disease between farms (and fields within farms). The model distinguishes between susceptible, infested and symptomatic fields. It has been successfully tested against data for the spread of rhizomania in the UK between 1987 and 2002 (Stacey *et al*. 2004). The interacting life cycles of the crop, pathogen and vector are complicated, with seasonal forcing and amplification rate dependent upon soil type. Details of the construction, testing, simplification and parameterization of the model are given by Webb (1997), Truscott *et al*. (1997, 2000), Webb *et al*. (1997, 2000), Truscott & Gilligan (2001, 2003) and Stacey *et al*. (2004). In brief, the amplification of inoculum is described by the following set of differential equations:

$$\begin{array}{ll} \text{Total}\;\text{roots} & \frac{\text{d}N}{\text{d}t}=r(1-N),\\ \text{Susceptible}\;\text{roots} & \frac{\text{d}S}{\text{d}t}=r(1-N)-\lambda (T)M_{\text{i}}-mS,\\ \text{New}\;\text{inoculum} & \frac{\text{d}M}{\text{d}t}=Q\lambda (T)M_{\text{i}}S. \end{array}$$

The parameter, *λ*(*T*), is a temperature-sensitive force of infection/unit inoculum and *M*_i_ is the initial concentration of inoculum in the vicinity of a plant, measured in infectious units per 100 g soil. The parameters *r*, *m* and *Q* represent root growth rate, the inverse of the susceptible period for the roots and the amplification of inoculum within infected root tissue, respectively. There is a threshold density for inoculum, *M*=*M*_c_ at the end of the season for expression of symptoms (Tuitert & Hofmeester 1992). The temperature sensitivity of the force of infection is represented by a switching mechanism,

$$\lambda (T)=\{\begin{array}{c} \lambda_{m},\;T\geq T_{\text{c}}\\ 0,\;T<T_{\text{c}} \end{array}$$

where *T* is the soil temperature and *T*_c_=12°C is a critical temperature for infection and amplification of inoculum (Webb *et al*. 2000; Truscott & Gilligan 2003). The temperature of the soil is a function of insolation and also the soil type in which the crop is growing. Heavier and organic soils heat up more slowly than light ones and hence retard the development of the disease. Details of the derivation of the amplification model, which represents a simplification of a more complicated model, are given by Truscott *et al*. (2000) and Stacey *et al*. (2004). The *r*, *m* and *Q* parameters for the amplification model were estimated by fitting to data from disease progress experiments (Blunt *et al*. 1992; Tuitert 1994). Details of the soil–temperature dependence are given by Stacey *et al*. (2004). In brief, data for seven different classes of soil type modulated by insolation (obtained from MAFF) were input to the model on a granularity value of 1 km: the parameter *λ*_*m*_, and hence *λ*(*T*), were linearly scaled according to soil type–climate class and parameterized from published data (Blunt *et al*. 1991; Tuitert 1994; Tuitert & Hofmeester 1994). The principal qualitative results from the model presented below are robust to fine detail concerning temperature and soil insolation. The default parameters are summarized in table 1.

The dispersal phase is modelled on three hierarchical spatial scales: within a field; between fields on a farm; and between farms at a regional or national level. A small number of sites, each comprising three or four randomly selected fields, represent the initial introduction of inoculum from outside the UK. Movement of inoculum within a field by machinery is described by a redistribution kernel (Truscott & Gilligan 2001),

$$p(r)\text{d}r=\{\begin{array}{ll} (1-p)\delta (r)+pk\;\text{exp}(-kr), & r>0\\ 0, & \text{otherwise} \end{array}$$

where *p*(*r*) is the probability density of a unit of inoculum being moved a distance *r* in the direction of travel. Applying this to the distribution of inoculum, *M*_i_ at the end of a season gives the distribution for the start of the next season. The parameters of the distribution were estimated from aerial photographs of symptomatic fields supplied by MAFF (table 1). The spatial patterns produced by this model accurately reproduced those observed in the field (Truscott & Gilligan 2001). Transmission within farms occurs from infested fields to a Poisson-distributed number of recipient fields (with mean 4.5, obtained by inspection of farm size and empirical outbreak data supplied by Defra) when beet is harvested in the donor field. An infested field is considered infectious only in a year in which beet is grown and harvested from that field. The amount of inoculum transmitted is proportional to the total inoculum, *M*_i_, in the infectious field. At the national level, all farms have a Poisson-distributed number of contact farms (with mean 16) with which they share machinery and risk infesting with inoculum. These farms are chosen from within a maximum radius of 10 km. The limit of 10 km was arbitrarily defined following exploration of simulations involving a range of radii, informed by the patterns of outbreaks on farms early in the rhizomania epidemic. An infectious farm (one which contains at least one infested field) is the source of a Poisson-distributed number of visits to its contacts, each of which causes infestation in the recipient. In addition, all transmission processes are dependent on the rotation period of the beet crop. Beet is grown with a 3- to 4-year rotation period with other crops. Transmission parameters for within and between farms were fitted, using maximum likelihood, to outbreak data for the UK, provided by MAFF. In addition to these internal sources of infestation, we include an external source to represent the mechanism that initially introduced the disease. This source introduces new infestation foci into the country as a Poisson process over time (mean, 0.5 new sites per year), homogeneously distributed in space. This corresponds to the rate of appearance of new foci within the UK in the first 10 years of the epidemic (Stacey *et al*. 2004). Note that although the imposition of a 10 km limit prevents occasional long-distance farm-to-farm movements, the initiation of foci beyond 10 km is still possible owing to the arrival of external inoculum. Statistical measures were drawn from multiple realizations of the stochastic evolution of the model. The first 5 years of outbreak history in the UK were used as the initial conditions for simulations (Stacey *et al*. 2004). For each realization, the positions and quotas of the farms were randomly assigned. This was done at the county scale using data for numbers of growers and the distribution of sugar-beet quotas among growers within each county supplied by the industry representative, British Sugar PLC. The position of each grower was randomly assigned somewhere in a county along with a sugar-beet quota assigned according to the distribution of quotas for the county. The number of realizations used to calculate results is stated in the text and figure legends where appropriate.

### 2.2 Behaviour of growers

The model is used to compare the effectiveness of field-scale ‘containment’ policy with a farm-scale ‘stewardship’. For the field-scale strategy, sugar beet is no longer grown in an infested field once symptoms are detected in that field. We assume that growers will practice field-scale containment within the farm-scale strategy. We allow two degrees of freedom for the farm-scale stewardship policy. The first is the growers' awareness of the disease state of contacts with whom they share machinery. This can be used to vary the effective length-scale of the strategy: a zeroth-order strategy means the grower responds to disease on his or her own farm; first-order means a response to immediate contacts; second-order to contacts of contacts; and so on. Second, the growers' sensitivity to risk (0≤*π*≤1) is used as a measure of the tendency of growers to respond to the local risk of rhizomania. Risk aversion is modelled by the probability that an individual grower donates his or her quota to the stewardship scheme. It is given as

$$R=\{\begin{array}{c} \alpha \pi \xi ,\;\alpha \pi \xi <1\\ 1,\;\text{otherwise}. \end{array}$$

The parameter, *ξ*, represents the proportion of the response neighbourhood showing symptoms: where, for example, for a first-order strategy, the response neighbourhood is defined as the total number of sugar-beet growers with whom the candidate grower has direct contact by sharing machinery. The parameter, *α*, is the supremum of the set of response neighbourhood sizes over all growers. The stewarding parameter, *π*, is a measure of how responsive growers are to the presence of rhizomania within their response neighbourhood. The definition of *α* standardizes *π*, such that it corresponds to the grower with the largest response neighbourhood stewarding their crop with probability 1 on the discovery of a single symptomatic field in their response neighbourhood. There is a corresponding willingness to accept quota among growers that assesses the disease status of neighbouring farms.

### 2.3 Local deployment of partially resistant varieties

Finally, we consider the effect of local deployment of partially resistant varieties that reduce the strength of connections between infested and susceptible sites. Two parameters control the response: the reduction in amplification of inoculum (*Q*) by partial resistance and the growers' awareness of the disease status of contacts, as for the stewardship scheme.

## 3. Results

### 3.1 Field-scale containment

We examined the effectiveness of the field-scale response by running the model from the initial infestation up to 2000 for the East Anglian region with and without containment. The combination of the threshold for symptoms and crop rotation with periods generally greater than 3 years results in disease remaining undetected in beet-growing fields for more than a decade. As a result, soil infestation is much more extensive than symptoms indicate. It is clear that this field-scale approach to control fails to prevent invasion (figure 1). The model shows proliferation of disease around the three sites of initial infestation. This is supported by empirical observation of the continued spread of rhizomania in the UK during the period in which the containment policy was in place (Stacey *et al*. 2004). Large numbers of symptomatic fields have since been recorded in East Anglia (Anonymous 2006; M.J.C. Asher 2007, personal communication). Maps for the spread of symptomatic and infested fields show that a 20 km wide margin of asymptomatic infestation surrounds symptomatic patches (figure 1). This is approximately equivalent to two infectious transmissions beyond the symptomatic farms.

### 3.2 Farm-scale schemes

Three strategies are compared in figure 2 by allowing the model to run from 2000 to 2050. The zeroth-order farm-scale strategy fails even when all growers are obliged (*π*=1) to transfer quota, so does a weak response (*π*<0.1) to second-order contacts, but a strong response (*π*≥0.9) to second-order contacts succeeds in limiting the spread of disease.

Further analysis of the responsiveness to disease in neighbouring or contact farms shows the impact of scale on the effectiveness of control by redistribution of cropping quota (figure 3). The disease is best controlled when the farm-scale scheme matches the temporal and spatial scales of the disease (given as 20 km for the infectious disease front). The most effective scale of control occurs with a neighbourhood of size 2, which we now examine in more detail by considering a strain of the pathogen with enhanced amplification of inoculum. The dependence of disease control on the stewardship parameter divides into three phases (figure 3, inset). When *π* is sufficiently small, i.e. when growers show little sensitivity to risk, the disease escapes from being a small focus and spreads at its maximum rate. A large area becomes infested by the disease and the stewardship scheme therefore becomes saturated even though the stewardship parameter is small. When *π* is sufficiently large, stewardship reduces the force of infection due to an infectious site during its infectious lifetime and invasion is prevented, provided there is permanent blacklisting of symptomatic fields and hence removal from the system. In the middle phase, there is a large variance in the number of symptomatic farms as stewardship successfully controls the disease in only a proportion of the iterations.

### 3.3 Local deployment of partially resistant varieties

We consider three levels of resistance equivalent to 80, 99 and 100% reduction in amplification (figure 4). An 80% reduction, typical of currently available varieties (Scholten & Lange 2000), has only a small effect in reducing infestation, with almost 50% farms infested by 2050 (figure 4*a*). However, the spread of disease can be reduced by local deployment of highly resistant varieties (99% reduction in amplification) (figure 4*b*), but a substantial reduction in the proportion of infested farms (below 50%) occurs only when the neighbourhood of contact is greater than or equal to 2. The extreme case of using complete resistance (which is analogous to a stewardship parameter of *π*=1) shows that the reduction in disease increases asymptotically with neighbourhood of contact (figure 4*c*). This differs from the stewardship scheme, which is non-monotonic with respect to neighbourhood size owing to the additional constraint of redistributing susceptible crops in the landscape (figure 3).

## 4. Conclusions and discussion

Our results show the importance of matching the scales of local control strategies to prevent intensification and regional spread of disease with the inherent temporal and spatial scales of an epidemic, using rhizomania disease of sugar beet as a motivation for the analyses. We conclude that the disease could have been managed by movement of susceptible crops in the landscape using a strategy that takes account of risk aversion among growers, and we show parallels and differences between the use of a ‘culling’ strategy and the deployment of partially resistant varieties. A simple field-scale containment strategy, whereby the susceptible crop is no longer grown on fields showing symptoms, fails for this system with cryptic infection. By the time symptoms appear, usually after three or more cycles of beet cultivation, there has been sufficient time for amplification of inoculum and dispersal on cultivation machinery to neighbouring fields. The field-scale strategy therefore lags behind the epidemic. It fails because it underestimates both the temporal scale of the epidemic (given by amplification of inoculum on an infested site prior to detection) and the spatial scale (which reflects distance of transmission from the infested site to neighbouring fields). Shortening the time of response by allowing growers to react to the disease status of neighbours allows the containment of disease. This is analogous in principle to culling strategies for foot and mouth disease, in which culling is extended to contact as well as infected premises to allow for cryptic infection (Keeling *et al*. 2001, 2003; Tildesley *et al*. 2006). The farm-scale strategy also allowed us to analyse the effects of the degree of risk aversion among growers on the effectiveness of control.

We implemented the farm-scale strategy by allowing growers to exchange quotas for sugar-beet cultivation, so that the susceptible crop is moved through the landscape in response to local perception of disease risk. The scheme allows for flexibility in the growers' response to risk (encapsulated in *π*) and shows that there is a natural scale of response equivalent to two farms. This scale reflects the efficiency of local control subject to the global constraints of relocating the susceptibles within a dynamically changing landscape of available sites. When the neighbourhood for control is smaller than that into which the disease spreads prior to detection (e.g. neighbourhood of size 1), the stewardship scheme causes a reduction in disease incidence of at most 20% (figure 3). In contrast, when the neighbourhoods match, the disease incidence is reduced by four orders of magnitude for large values of the stewardship parameter, *π* (figure 3). When, however, the neighbourhood of control exceeds the characteristic scale of the disease, the efficiency of control diminishes. So much susceptible acreage is moved into the scheme that it becomes saturated; that is, the donated quota exceeds the amount that can be accommodated elsewhere in the country. This occurs because the larger control neighbourhood blocks asymptomatic fields from elsewhere in the country being accommodated at the cost of susceptibles being moved unnecessarily. Therefore, a neighbourhood of size 3 has a very similar effect to a neighbourhood of size 1 (figure 3).

Partially resistant crops reduce but do not stop the amplification of inoculum. This in turn reduces the transmission of viruliferous inoculum between infested and susceptible (i.e. uninfested) fields. It is analogous to a partially effective prophylactic drug. Unlike the stewardship strategy, there is no optimal scale for deployment. There is instead a monotonic decrease in infestation with increasing scale of local deployment. This behaviour occurs even when the plants are completely resistant to rhizomania since there is no need to find new sites for beet cultivation that gives rise to an optimal scale for the stewardship approach. The use of partially resistant varieties is not, however, without risk. A reduction in amplification of inoculum of 80% (equivalent to currently available varieties) does little to reduce infestation (figure 4). The pathogen continues to amplify inoculum and to spread, even with a 99% reduction. Successful management of disease therefore demands continued availability of resistant varieties. Withdrawal of partially resistant varieties, for example if those varieties were to prove sensitive to novel strains of another pest or pathogen, would expose the crop to large amounts of inoculum of beet necrotic yellow vein virus, built up during cultivation of the partially resistant varieties. The deployment of partially resistant varieties arguably therefore retains a longer term risk from rhizomania. Partially resistant varieties are not, of course, the only methods whereby additional management of the disease is possible. For emerging infections such as rhizomania, restrictions might also be introduced to reduce the transmission of inoculum between farms: obvious methods are improved disinfection of machinery and distance-related restrictions on movements of shared machinery, features that can be analysed using the current model. Changes in rotation frequency are also possible to alter the effective rate of amplification (Stacey *et al*. 2004).

We have used a rather complicated model to analyse the efficiency and dynamics of control in order to match the detail of a particular host–pathogen system while also addressing generic issues. The model had previously been parameterized and tested for rhizomania disease of sugar beet in the UK (Truscott *et al*. 1997, 2000; Webb 1997; Webb *et al*. 1997, 2000; Truscott & Gilligan 2001, 2003; Stacey *et al*. 2004). Rhizomania disease of sugar beet serves as an exemplar of a highly, spatially structured and partially asymptomatic epidemic that spreads over successive seasons through a crop landscape, which changes as crops are rotated around farms. It is, of course, possible to analyse the generic issues of control using simpler stochastic spatial models. We have described elsewhere how to optimize control of disease in small-world networks by matching the scale of local control, analogous to vaccination, with the natural scale of epidemics with cryptic infection (Dybiec *et al*. 2004; Kleczkowski *et al*. 2006), but without allowing for movement of susceptible crops through the landscape. Our current model uses a simple criterion for optimization, which is implicitly defined as the proportion of symptomatic (or infested) farms (cf. figure 3). This criterion does not take explicit account of the costs of disease nor of the costs of control. These can be included within an objective function for minimization. An example is given for a spatially explicit epidemic model based upon the contact process by Forster & Gilligan (2007), which holds for plant and animal disease.

Finally, we consider some parallels between the epidemiology and control of botanical and animal epidemics. Although modelling of botanical epidemics historically tended to develop independently of advances in animal and medical epidemiology, the commonality of the underpinning theory is now evident and widely accepted (Gilligan 2002; Burdon *et al*. 2006). This is especially true in considering the spatial dynamics of plant disease in which the unit of interest may be a field or an entire farm when considering regional spread. It leads naturally to considerations of compartmental models for the change in state from susceptible to infected and removed classes common to plant, animal and medical epidemiology (Gilligan 2002). There are also commonalities in mapping control strategies onto epidemiological models. Removal of infected and susceptible crops is analogous to culling practices in livestock systems. Prophylactic treatment of fields by fungicides renders susceptible crops temporarily resistant to infection in a manner analogous to vaccination. Similarly, eradicant fungicides reduce infectious periods like therapeutic drugs. But there are differences. One characteristic is the ability to rotate crops which changes the geometry of susceptible crops from season to season in a way that is different from many livestock systems. Genetical control, as described here, is widely used to manage plant disease. It is less common in controlling animal disease, but the net effects of partial disease resistance in plants translate readily into recognizable epidemiological strategies for reducing transmission rates or infectious periods. Complete genetical control is comparable to vaccination by effectively removing susceptible crops into the removed class. Space can be accommodated, as here, by a network or metapopulation models for spread through heterogeneous systems and by reaction–advection–diffusion and integro-differential equations for continuous homogeneous environments. Taylor *et al*. (1998) and Swinton & Gilligan (1999) used stochastic metapopulation models to identify strategies for the control Dutch elm disease and chestnut blight in semi-natural environments. Stochastic metapopulation, percolation and network models have subsequently been used to analyse conditions for the invasion and persistence of pathogens in spatially explicit crop populations (Park *et al*. 1999, 2003; Gibson *et al*. 2006), for the evolution of virulence and resistance in host–pathogen systems, mostly in semi-natural environments (Antonovics *et al*. 1997; Thrall & Burdon 2002, 2003; Burdon *et al*. 2006), the evolution of fungicide resistance at local and national scales (Parnell *et al*. 2005, 2006) and the deployment of local control for cryptic infection of plant pathogens (Dybiec *et al*. 2004). With the exception of work on disease of semi-natural populations (Antonovics *et al*. 1997; Burdon *et al*. 2006), however, few of these address examples with truly realistic spatial heterogeneities, the subject of this paper.

## Figures and Tables

**Figure 1:**
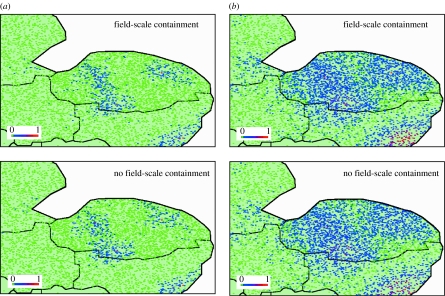
Estimated spread of rhizomania disease in the East Anglian region by the year 2000 in the presence and absence of a field-scale containment policy (initiated in 1979). Susceptible farms are shown in green. The intensity of blue–red shading indicates the probability of a site being in a particular state ((*a*) symptomatic/(*b*) infectious) ranging from blue (negligible) to red (1). Probabilities were estimated from 50 iterations of the model. The results show that field-scale containment fails and the advance of a region of infested sites ahead of the symptomatic sites.

**Figure 2:**
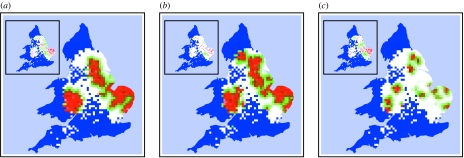
Effect of stewardship on projected rhizomania disease incidence in 2050 (means of 20 iterations). The colours denote no beet grown (dark blue), no rhizomania risk (white), rhizomania infested but not symptomatic (green), mix of symptomatic and infested farms (brown) and symptomatic (red) with intensity of colour corresponding to the proportion of farms infested or symptomatic. Each square corresponds to 10 km. Insets show areas donating quota to the stewardship scheme (red) and receiving quota from the scheme (green) with intensity of colour denoting relative amount of quota donated or received per unit area. (*a*) Neighbourhood of size 0; growers respond by stewarding as much as possible. (*b*) Neighbourhood of size 2, weak response. Stewardship occurs dependent on state of neighbours, with high reluctance to steward (*π*=0.1). (*c*) Neighbourhood of size 2, strong response. Stewardship occurs dependent on state of neighbours, with high tendency to steward (*π*=0.9).

**Figure 3:**
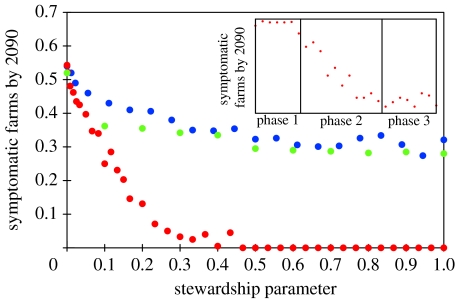
Interaction between neighbourhood size and tendency to steward. Proportion of symptomatic farms in 2090 for neighbourhood of sizes 1 (blue), 2 (red) and 3 (green) (means of 20 iterations). Optimum control is achieved when the neighbourhood matches the time and space scale of the epidemic (approx. neighbourhood of size 2). For stewarding with neighbourhood of size 1, the policy has a relatively small impact on rhizomania incidence by 2090, with at most a 20% reduction in symptomatic area. Similarly, for stewarding with neighbourhood of size 3, relatively little reduction in incidence is observed as the stewardship scheme becomes limited by the availability of uninfested land into which quota could be donated. The inset shows the equivalent diagram for an aggressive pathotype of rhizomania, which amplifies an order of magnitude more rapidly, with stewardship neighbourhood matched to the scale of the disease. The graph illustrates three-phase dependence of disease spread on stewarding, whereby the epidemic is insensitive to stewarding parameter in phases 1 and 3, and highly responsive in phase 2.

**Figure 4:**
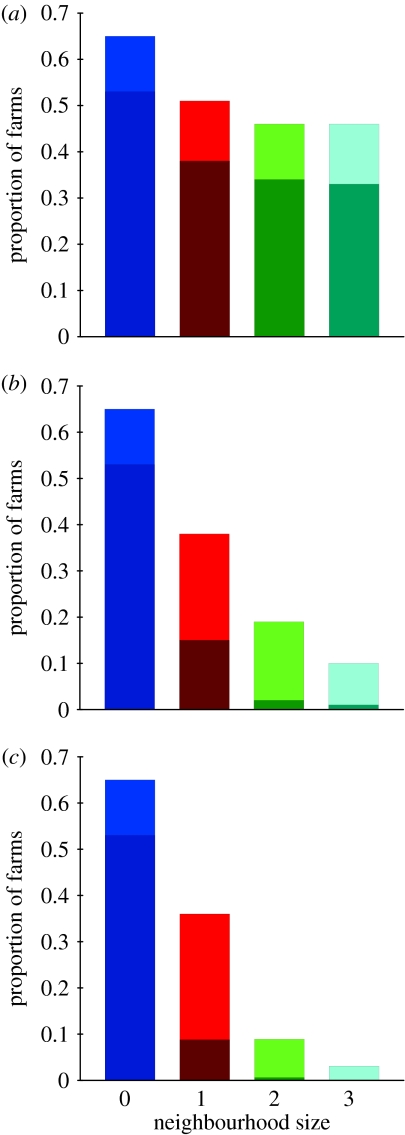
Proportion of farms infested (whole bar) and symptomatic (darker section) in 2090 for partially resistant varieties causing (*a*) 80% reduction in inoculum returned to the soil relative to susceptible varieties, (*b*) 99% reduction and (*c*) 100% reduction (fully resistant). Resistant varieties are deployed in response to rhizomania symptoms within the given response neighbourhoods (*x*-axes). The results shown correspond to 20 iterations.

**Table 1:** Default parameter values used in simulations.

description	value	units^a^
*amplification*		
growth rate (*r*)	0.1	d^−1^
(susceptible period for root)^−1^ (*m*)^−1^	0.07	d^−1^
force of infection (*λ*_*m*_)	0.84	(d.IU/100 g)^−1^
amplification (*Q*)	2.37	(d.IU/100 g)^−1^
inoculum threshold for symptom expression (*M*_c_)	0.066	IU/100 g
mean dispersal in field	0.2	m^−1^
*transmission*		
mean number of contacts	16	—
contact radius	10	km
mean number of farm visits	4	—
mean number of field visits	0.3	—
mean external infection rate	0.5	yr^−1^
*farm structure*		
no. beet-growing farms	15 000^b^	—
no. beet-growing fields/farm (±s.d.)	14 (±8)	—
no. fields sown to beet	20 000	
no. fields available for growing beet	200 000	—
*quota distribution*		
total area of sugar beet	150 000	ha
total yield of sugar beet	9 000 000	tonnes
average yield/field	450	tonnes
quota sizes (±s.d.)	180 (±0.65)	tonnes
average yield/field	450	tonnes

a IU, infectious unit of soil-borne inoculum.

b Distributed among 8500 growers allowing for multiple ownership.
